# Brain perfusion in fibromyalgia patients and its differences between responders and poor responders to gabapentin

**DOI:** 10.1186/ar3673

**Published:** 2012-02-09

**Authors:** Chie Usui, Kotaro Hatta, Nagafumi Doi, Atsushi Nakanishi, Hiroyuki Nakamura, Kusuki Nishioka

**Affiliations:** 1 Department of Psychiatry, Juntendo University School of Medicine, 2-1-1 Hongo, Bunkyo-Ku, Tokyo 113-8421, Japan; 2 Ibaraki Prefectural Tomobe Hospital, 654 asahi-cho, kasama-city, Ibaraki 309-1717, Japan; 3 Department of Radiology, Juntendo University School of Medicine, 2-1-1 Hongo, Bunkyo-Ku, Tokyo 113-8421, Japan; 4 Department of Environmental and Preventive Medicine, Graduate School of Medical Science, Kanazawa University, kakuma-cho, Kanazawa-city, Kanazawa 920-1192, Japan; 5 Institute of Innovative Medical Science and Education, Tokyo Medical University, 6-1-1 Shinjyuku, Shinjyuku-ku, Tokyo 160-8402, Japan

## Purpose

The aim of the present study was to determine the brain areas associated with fibromyalgia, and whether pretreatment regional cerebral blood flow (rCBF) can predict response to gabapentin treatment.

## Methods

A total of 29 women with fibromyalgia and 10 healthy women without pain matched for age were finally enrolled in the study. Technetium-99 m ethyl cysteinate dimer single photon emission computed tomography (^99 m^Tc-ECD SPECT) was performed in the fibromyalgia patients and controls. A voxel-by-voxel group analysis was performed using SPM2. After treatment with gabapentin, 16 patients were considered "responders", with decrease in pain of greater than 50% as evaluated by visual analogue scale (VAS). The remaining 13 patients were considered "poor responders".

## Results

**Figure 1:**
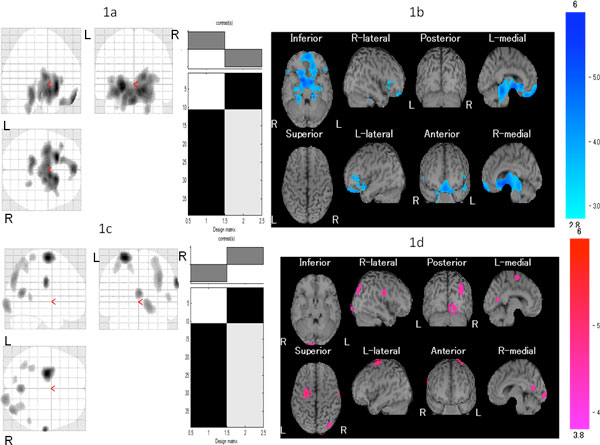
**Comparison of rCBF between patients with FM and age-matched healthy controls**. Maximum intensity projections of SPM2 results from comparison of rCBF between patients with FM and age-matched healthy controls. **a, b **The FM patient group exhibited significant hypoperfusion in the left culmen. **c, d **The FM patient group exhibited significant hyperperfusion in the right precentral gyrus, right posterior cingulate, right superior occipital gyrus, right cuneus, left inferior parietal lobule, right middle temporal gyrus, left postcentral gyrus, and left superior parietal lobule. Height threshold is < 0.001, corrected for multiple comparison.

Compared to control subjects, we observed rCBF abnormalities in fibromyalgia including hypoperfusion in the left culmen and hyperperfusion in the right precentral gyrus, right posterior cingulate, right superior occipital gyrus, right cuneus, left inferior parietal lobule, right middle temporal gyrus, left postcentral gyrus, and left superior parietal lobule (Table 1, Figure 1). Compared to responders, poor responders exhibited hyperperfusion in the right middle temporal gyrus, left middle frontal gyrus, left superior frontal gyrus, right postcentral gyrus, right precuneus, right cingulate, left middle occipital gyrus, and left declive (Table 2). The right middle temporal gyrus, left superior frontal gyrus, right precuneus, left middle occipital gyrus, and left declive exhibited high positive likelihood ratios.

**Table 1:** Regions of significant hyperperfusion and hypoperfusion in the FM group.

	κ	Z score	x(mm)	y(mm)	z(mm)	Localisation
**Hyperperfusion**	134	4.55	66	-10	30	R **Precentral Gyrus**
	262	4.16	2	-62	14	R **Posterior Cingulate**
	824	3.98	36	-82	32	R **Superior Occipital Gyrus**
	429	3.95	18	-96	-6	R **Cuneus**
	220	3.57	50	-38	52	L **Inferior Parietal Lobule**
	55	3.54	52	-46	6	R **Middle Temporal Gyrus**
	113	3.52	-30	-42	68	L **Postcentral Gyrus**
		3.74	-14	-74	56	L **Superior Parietal Lobule**
	709	4.66	-2	56	-22	L **Superior Frontal Gyrus**
**Hypoperfusion**	1111	4.38	-12	-32	-18	L **Culmen**

Results are listed by clusters.κvalue, Z score, Talairach coordinates of peak voxel, and anatomic localization are provided for each cluster.

**Table 2:** Regions of significant hyperperfusion in the poor responder group compared to the responder group.

	κ	Z score	x(mm)	y(mm)	z(mm)	Localisation
**Hyperperfusion**	1260	4.08	42	-62	16	R **Middle Temporal Gyrus**
	95	3.88	-46	6	50	L **Middle Frontal Gyrus**
	95	3.88	-20	38	52	L **Superior Frontal Gyrus**
	69	3.67	56	-12	56	R **Postcentral Gyrus**
	578	3.67	14	-76	28	R **Preuneus**
	59	3.58	4	20	36	R **Cingulate**
	70	3.54	-20	-80	4	L **Middle Occipital Lobule**
	77	3.51	-20	-80	-26	L **Declive**

Results are listed by clusters. κvalue, Z score, Talairach coordinates of peak voxel, and anatomic localization are provided for each cluster.

## Conclusion

The present study revealed brain regions with significant hyperperfusion associated with the default-mode network, in addition to abnormalities in the sensory dimension of pain processing and affective-attentional areas in fibromyalgia patients. Furthermore, hyperperfusion in these areas was strongly predictive of poor response to gabapentin.
